# Sugammadex, neostigmine, and postoperative pulmonary complications: protocol of the SNaPP multicentre randomised controlled trial

**DOI:** 10.1016/j.bjao.2026.100531

**Published:** 2026-02-10

**Authors:** Kate Leslie, Sabine Braat, Jai N. Darvall, Philip J. Peyton, Nancy Devlin, Paul S. Myles, Tomás B. Corcoran, Timothy G. Short, Matthew T.V. Chan, Benjamin L. Olesnicky, An Tran-Duy, Helen Maxwell-Wright, Cindy Schultz-Ferguson, Sofia Sidiropoulos, David A. Story

**Affiliations:** 1 Department of Critical Care, Melbourne Medical School, University of Melbourne, Melbourne, Victoria, Australia; 2 Department of Anaesthesia and Pain Management, Royal Melbourne Hospital, Melbourne, Victoria, Australia; 3 Centre for Epidemiology and Biostatistics, Melbourne School of Population and Global Health, University of Melbourne, Melbourne, Victoria, Australia; 4 Methods and Implementation Support for Clinical Health (MISCH) Research Hub, Faculty of Medicine, Dentistry, and Health Sciences, University of Melbourne, Melbourne, Victoria, Australia; 5 Department of Intensive Care, Royal Melbourne Hospital, Melbourne, Victoria, Australia; 6 Department of Anaesthesia, Austin Hospital, Melbourne, Victoria, Australia; 7 Centre for Health Policy, Melbourne School of Population and Global Health, University of Melbourne, Melbourne, Victoria, Australia; 8 Department of Anaesthesiology and Perioperative Medicine, School of Translational Medicine, Faculty of Medicine, Nursing and Health Sciences, Monash University, Melbourne, Victoria, Australia; 9 Department of Anaesthesiology and Perioperative Medicine, The Alfred, Melbourne, Victoria, Australia; 10 School of Medicine and Pharmacology, Faculty of Health and Medical Sciences, University of Western Australia, Perth, Western Australia, Australia; 11 Department of Anaesthesia and Pain Medicine, Royal Perth Hospital, Perth, Western Australia, Australia; 12 Department of Anaesthesiology, University of Auckland, Auckland, Aotearoa New Zealand; 13 Department of Anaesthesia, Auckland City Hospital, Auckland, Aotearoa New Zealand; 14 Department of Anaesthesia and Intensive Care, Faculty of Medicine, Chinese University of Hong Kong, Hong Kong SAR, China; 15 Department of Anaesthesia and Intensive Care, Prince of Wales Hospital, Hong Kong SAR, China; 16 Sydney Medical School, Faculty of Medicine and Health, University of Sydney, Sydney, New South Wales, Australia; 17 Department of Anaesthesia, Royal North Shore Hospital, Sydney, New South Wales Australia

**Keywords:** abdominal surgery, anaesthesia, neostigmine, postoperative pulmonary complications, randomised controlled trial, sugammadex, thoracic surgery

## Abstract

**Background:**

Sugammadex, a novel cyclodextrin reversal agent, reduces the incidence of residual neuromuscular block compared with neostigmine and was associated with fewer postoperative pulmonary complications in a systematic review of small randomised trials. However, evidence from a large RCT is required.

**Methods:**

We designed an international, multicentre RCT (the SNaPP study). A total of 3500 patients, aged ≥40 yr and undergoing abdominal or thoracic surgery, will be enrolled and randomly allocated in a 1:1 ratio to receive sugammadex or neostigmine for reversal of neuromuscular block, stratified by centre. The primary outcome is a composite of postoperative pulmonary complications (atelectasis, pneumonia, acute respiratory distress syndrome, aspiration pneumonitis, or a combination of these) or death until hospital discharge (or postoperative day 7 if still in hospital). Secondary outcomes are components of the primary outcome, postoperative nausea and vomiting, unplanned ICU/high-dependency unit admission, days alive and at home at 30 days, and health-related quality of life at 3 months. The trial aims to determine whether sugammadex compared with neostigmine reduces the incidence of postoperative pulmonary complications or death. Data will be analysed on an intention-to-treat basis.

**Ethics and dissemination:**

The SNaPP study is approved by the ethics committees at participating sites in Australia, Aotearoa New Zealand, and Hong Kong and is endorsed by the Australian and New Zealand College of Anaesthetists Clinical Trials Network. Participant recruitment began on 20 July 2023 and was completed on 3 July 2025. Publication of the SNaPP study results is anticipated in 2026.

**Clinical trial registration:**

ACTRN 12623000394640.

## Postoperative pulmonary complications

Postoperative pulmonary complications are common, serious, costly, and distressing for patients.^1^ The incidence of postoperative pulmonary complications varies widely, depending on the definition and population, but many studies in noncardiac surgery estimate the incidence at more than 10%.^2^, ^3^, ^4^, ^5^, ^6^, ^7^, ^8^, ^9^ Postoperative pulmonary complications are associated with increased length of hospital stay,^10^^,^^11^ mortality,^11^^,^^12^ and cost.^5^^,^^10^^,^^13^ In a multicentre Spanish study, postoperative pulmonary complications were associated with longer median length of hospital stay (12 days *vs* 3 days) and higher 30-day (19.5% *vs* 0.5%) and 90-day (24.4% *vs* 1.2%) mortality.^11^ In a large database study from the USA, postoperative pulmonary complications were associated with a 50% increase in the cost of an admission.^5^ A systematic review revealed that postoperative pulmonary complications adversely affect long-term quality of life and cause physical, emotional, and social distress.^14^

Postoperative pulmonary complications result from pathophysiological changes associated with anaesthesia and surgery, including pulmonary alveolar collapse and microbial contamination.^1^^,^^15^^,^^16^ General anaesthesia impairs respiratory control, respiratory muscle activity, and alveolar microcilial and surfactant function, causing pulmonary collapse. In addition, increased inspired oxygen concentrations promote absorption atelectasis. These changes are exacerbated by intermittent positive pressure ventilation, reduced cardiac output secondary to anaesthetics, and direct and indirect compression of lung tissue during surgery.^1^^,^^15^^,^^16^ General anaesthesia is also associated with an increased risk of airway contamination which may arise from impaired pharyngeal and upper oesophageal reflexes.^17^ After surgery, residual effects of anaesthetics and neuromuscular blocking drugs, administration of opioids, and systemic inflammatory responses further impair hypoxic drive, ventilation, and upper airway patency. Pulmonary atelectasis, pneumonia, aspiration pneumonitis, and acute respiratory distress syndrome may result, particularly in older patients having abdominal or thoracic surgery.^1^^,^^15^^,^^16^

## Reversal of neuromuscular block

Non-depolarising neuromuscular blocking drugs are administered during general anaesthesia to facilitate tracheal intubation, mechanical ventilation, and surgery. Traditionally, non-depolarising neuromuscular blocking drugs have been reversed with an anticholinesterase (e.g. neostigmine), together with an anti-muscarinic agent (e.g. glycopyrrolate) to counter unwanted muscarinic effects. More recently sugammadex, a novel cyclodextrin encapsulating agent, has become available for reversal of rocuronium and vecuronium. A systematic review concluded that sugammadex achieves complete recovery from neuromuscular block more rapidly than neostigmine (2.2 min *vs* 12.0 min, mean difference 10.2 min, 95% confidence interval [CI] 8.5–12.0 min) but does not abolish residual neuromuscular block altogether (4.8% *vs* 16.0%, risk ratio [RR] 0.40, 95% CI 0.28–0.57).^18^ Nevertheless, a strategy utilising sugammadex will likely be more effective at reducing the incidence of residual neuromuscular block than one using neostigmine. However, despite the demonstrated reduction in the incidence of residual neuromuscular block with sugammadex, the evidence for a benefit of sugammadex with respect to postoperative pulmonary complications is inconclusive.

A large retrospective cohort study (*n*=45 712) reported that sugammadex was associated with fewer postoperative pulmonary complications than neostigmine at hospital discharge (adjusted odds ratio [OR] 0.70, 95% CI 0.63–0.77).^19^ In contrast, another large retrospective cohort study (*n*=83 250) reported no difference in a composite of post-extubation oxygen desaturation, respiratory failure requiring noninvasive ventilation, or tracheal re-intubation within 7 days (adjusted OR 1.01, 95% CI 0.94–1.08).^20^ A large prospective cohort study (*n*=8795) also reported that sugammadex was not associated with fewer postoperative pulmonary complications than neostigmine at 28 days (adjusted OR 1.03, 95% CI 0.85–1.25).^21^ In a recent systematic review of randomised trials reporting postoperative pulmonary complications beyond 24 h after surgery, the OR was 0.67 (95% CI 0.47–0.96) for postoperative pulmonary complications (13 trials, 1307 patients) with sugammadex compared with neostigmine.^22^ A large RCT therefore is justified.

## Rationale for study design

We will include patients having elective or expedited abdominal or intrathoracic surgery lasting ≥2 h, because these patients are at risk of postoperative pulmonary complications based on age,^11^^,^^23^ smoking,^24^ comorbidity,^25^ anaemia,^11^ surgical incision,^26^ and duration of surgery.^27^ The results of a large trial using this cohort will be generalisable to tens of millions of patients worldwide.

We designed a pragmatic trial aimed at providing evidence on the comparative effectiveness of sugammadex and neostigmine in routine clinical practice.^28^ Attending anaesthetists are encouraged, but not mandated, to follow guidelines about quantitative neuromuscular monitoring, reversal agent doses, and timing of reversal of neuromuscular block and tracheal extubation.^29^, ^30^, ^31^, ^32^ Apart from the administration of rocuronium or vecuronium, and the randomised intervention, all other aspects of perioperative care are at the discretion of the treating team.

Participants are randomised shortly after induction of general anaesthesia, to ensure that participants are included in the intention-to-treat population. We advise the attending anaesthetists about the randomisation close to surgical wound closure, to ensure that patient care during surgery is not influenced by the treating team knowing the identity of the randomised intervention. On this basis, we advise anaesthetists to aim for reversibility of neuromuscular block by neostigmine at the end of surgery. We assumed that, if anaesthetists are made aware of the randomisation at the beginning of surgery, they might aim for deeper neuromuscular block in the sugammadex group than in the neostigmine group. The success of this approach will be assessed by comparing neuromuscular blocking drug doses between the two groups.

We use the Standardised Endpoints for Perioperative Medicine - Core Outcome Measures in Perioperative and Anaesthetic Care (StEP-COMPAC) definition of postoperative pulmonary complications.^33^ This definition is a composite of four diagnoses that are mechanistically linked to anaesthesia via airway collapse and contamination: atelectasis, pneumonia, acute respiratory distress syndrome, and aspiration pneumonitis. Our primary outcome is a composite new pulmonary complications or death to avoid a competing risk posed by death. Death up to hospital discharge (or postoperative day 7 if still in hospital) is expected to be rare in this patient population.

We assessed the feasibility of our trial with a survey of anaesthetists^34^ and a pilot feasibility study.^35^ In the survey, 147 (89%) respondents believed that postoperative pulmonary complications are important and 119 (72%) were willing to have their patients randomised. Among 150 eligible patients for our pilot feasibility trial, 120 consented to participate (recruitment rate 80%, 95% CI 73–86%). The mean (range) age of consented patients was 64 (41–83) yr, 70 (58%) were male and 50 (42%) were female, and planned surgeries were thoracic (23 [19%]), upper abdominal (41 [34%]), and lower abdominal (56 [47%]). The randomised intervention was administered without crossover to 115 of 117 patients who received reversal (98.3%, 95% CI 94.0–100%). Four of the 120 patients were lost to follow-up at 3 months (3.3%, 95% CI 0.9–8.3%).

## Methods

### Governance and operations

A completed Standard Protocol Items: Recommendations for Interventional Trials^36^ checklist is attached (Supplementary Appendix 1). The study was endorsed by the Australia and New Zealand College of Anaesthetists Clinical Trials Network on 17 November 2020. Participant recruitment began on 20 July 2023 and was completed on 3 July 2025 (23 months and 14 days). The trial project office is located in the Department of Critical Care, Melbourne Medical School, University of Melbourne, Melbourne, Australia. The trial steering committee, which includes two community members, oversees all aspects of the trial.

### Study design

The SNaPP study is a large (*n*=3500) international, multicentre, assessor- and patient-blinded RCT with patients randomised to sugammadex (intervention group) or neostigmine (control group) to reverse neuromuscular block at the end of abdominal and thoracic surgery. Group allocation is stratified by centre.

### Study hypothesis

Our primary hypothesis is that, compared with neostigmine, reversing neuromuscular block with sugammadex reduces the incidence of a composite new pulmonary complications or death up to hospital discharge (or postoperative day 7 if still in hospital) after general anaesthesia for abdominal and thoracic surgery in patients aged ≥40 yr.

### Participants and enrolment

We screened for patients aged ≥40 yr undergoing elective or emergency abdominal or thoracic surgery lasting ≥2 h and with an anticipated postoperative length of hospital stay of one or more night. Participant inclusion and exclusion criteria are listed in Box 1.

Box 1 **Inclusion and exclusion criteria**

**Inclusion criteria**

1. Aged 40 yr and older.
2. Plan for elective or expedited intraabdominal, retroperitoneal, pelvic, and noncardiac intrathoracic surgery.
3. Plan for relaxant general anaesthesia with an tracheal tube.
4. Surgery expected to last ≥2 h.
5. Expected hospital stay of one or more postoperative night.

**Exclusion criteria**

1. Unable to provide written informed consent (e.g. language barrier, intellectual disability, cognitive deficit, urgent surgery).
2. Plan for skin incision, vascular access, or both at or below the inguinal ligament without an abdominal or thoracic skin incision.
3. Plan for intraoperative administration of neuromuscular blocking drug other than rocuronium and vecuronium.
4. Plan to reverse neuromuscular block during surgery.
5. Plan to allow spontaneous complete recovery from neuromuscular block during surgery.
6. Contraindication to sugammadex or neostigmine.
7. Plan for elective postoperative invasive ventilation.
8. Previously randomised to the trial.

Alt-text: Box 1

The randomisation list was computer-generated by an independent statistician using randomly permuted blocks. On the day of surgery, and after successful induction of anaesthesia, an unblinded observer accesses the web-based randomisation portal. Participants are randomly assigned on a 1:1 basis to sugammadex or neostigmine, with stratification by site. The unblinded observer provides the randomisation results to the attending anaesthetist just before wound closure.

### Perioperative management

The participant timeline is presented in Table 1. Preoperative and postoperative management are at the discretion of the treating team. Most aspects of intraoperative management are at the discretion of the attending anaesthetist, including analgesic, anaesthetic, and antiemetic drugs; i.v. fluids; antibiotics; airway management and ventilation; and monitoring. Attending anaesthetists are encouraged, but not mandated, to follow guidelines recommending the use of quantitative neuromuscular monitoring to achieve adequate muscle relaxation during surgery and complete reversal of neuromuscular block before tracheal extubation. Neuromuscular block is achieved with rocuronium, vecuronium, or both. Succinylcholine is allowed at induction, if indicated. The randomised intervention is administered during or after surgical wound closure in a dose that is determined by the attending anaesthetist. Preparations of sugammadex and neostigmine that are available at participating sites are used. If further reversal is required, anaesthetists can use the reversal agent of their choice, although further doses of the randomised agent are encouraged. If a decision is made during surgery to reverse neuromuscular block during surgery or after the patient has left the operating room, the randomised intervention is not administered (and the anaesthetist administers the reversal agent of their choice). All randomised patients remain in the group to which they were assigned until the final assessment, on an intention-to-treat basis, unless the patient or their medical decision maker withdraws consent.

**Table 1:** Participant timeline. ∗Until hospital discharge or postoperative day 7 whichever is earlier.

Assessment	Baseline	Day 0	Day 1	Day 2	Day 3	Discharge	Day 30	3 months
Eligibility	x							
Consent	x							
Contact details	x							
Patient characteristics	x							
Medical history	x							
Quality of Recovery-15 score	x		x					
EQ-5D-5L score	x		x				x	x
Clinical Frailty Scale score	x							x
Randomisation		x						
Intraoperative data		x						
Administration of interventions		x						
Postoperative data		x						
Postoperative pulmonary complications∗		x	x	x	x	x		
Safety outcomes∗		x	x	x	x	x		
Postoperative nausea and vomiting			x					
ICU/high-dependency unit admission						x		
Days alive and at home							x	
Adverse events		x	x	x	x	x	x	x

### Data collection and auditing

Patients and research staff collecting postoperative data are blind to group allocation. The unblinded observer assists with unblinded data collection and database entry, and randomising the patient and advising the attending anaesthetist. Data are collected from the patient and their medical record at baseline, on the day of surgery, on postoperative days 1–3, at discharge (or postoperative day 7 if still in hospital), 30 days, and 3 months. There are no trial-specific investigations. Data relevant to the risk of postoperative pulmonary complications, including patient characteristics and major co-morbidities used in the Assess Respiratory Risk in Surgical Patients in Catalonia score,^11^ is collected before surgery, along with baseline measurements of the Quality of Recovery-15,^37^^,^^38^ EQ-5D-5L,^39^ and Clinical Frailty Scale^40^ scores. Details of surgery, anaesthesia, reversal, and tracheal extubation are collected, including doses of neuromuscular blocking drugs and reversal agents, use of quantitative neuromuscular monitoring, neuromuscular monitoring results at reversal and extubation, the time that the anaesthetist was made aware of the randomisation, that the trial intervention was administered, and that the tracheal tube was removed. Data relevant to the primary and secondary outcomes are recorded, including the duration of PACU stay, ICU/high-dependency unit stay, and hospital stay, and the results of Quality of Recovery-15, EQ-5D-5L, and Clinical Frailty Scale measurement. Safety outcomes and adverse events are also recorded.

Data are collected on a paper case report form and then entered onto a central electronic database that was designed, constructed, and maintained for the purposes of the trial. Identifying data remain at participating sites and are appropriately secured. Monitoring visits are conducted to assess the accuracy and legitimacy of the trial data and to determine compliance with Good Clinical Practice.

### Data safety and outcomes monitoring

An independent data safety and monitoring committee has been established. Experienced clinician scientists with expertise in perioperative medicine and an independent statistician are included. The committee’s charter outlines its roles, including reviewing and providing advice on the protocol, reviewing and interpreting accruing data, and reviewing and interpreting adverse outcomes to ensure participant safety. The committee has met three times, reviewing the results of the planned interim analyses at its third meeting, and recommending continuation of the trial as planned.

### Endpoint adjudication

An independent endpoint adjudication committee has been established. Experienced clinician scientists with expertise in assessing adverse pulmonary events are included. The committee’s terms of reference outline its role in adjudicating the primary endpoint of the trial, based on trial data and source documents accessed through a module in the trial database. Endpoint adjudicators are blind to group assignment. The manual of operations for the committee outlines how decision-making is escalated if the diagnosis of one or more pulmonary complications is uncertain. Decisions are completely independent from the trial coordinating team and steering committee.

### Trial outcomes

The primary outcome is a composite of new pulmonary complications or death up to hospital discharge (or postoperative day 7 if still in hospital), using the StEP-COMPAC definition (Box 2). ^33^ Death is defined as death from all causes. Secondary (which include the components of the primary outcome) and exploratory outcomes are listed in Box 3.

Box 2 **Standardised Endpoints for Perioperative Medicine - Core Outcome Measures in Perioperative and Anaesthetic Care definition of postoperative pulmonary complications33**

**Mechanism**

Composite of respiratory diagnoses that share common pathophysiological mechanisms including pulmonary collapse and airway contamination.

1. Atelectasis detected on computed tomography or chest radiograph.
2. Pneumonia using United States Centers for Disease Control criteria.^41^
3. Acute respiratory distress syndrome using Berlin consensus definition.^42^
4. Pulmonary aspiration (clear clinical history and radiological evidence).

**Severity**

1. None: planned use of supplemental oxygen or mechanical respiratory support as part of routine care, but not in response to a complication or deteriorating physiology. Therapies that are purely preventive or prophylactic, such as high-flow nasal oxygen or continuous positive airway pressure, should be recorded as none.
2. Mild: therapeutic supplemental oxygen <0.6 fraction inspired O_2_.
3. Moderate: therapeutic supplemental oxygen ≥0.6 fraction inspired O_2_, requirement for high-flow nasal oxygen, or both.
4. Severe: unplanned noninvasive mechanical ventilation, continuous positive airway pressure, or invasive mechanical ventilation requiring tracheal intubation.

**Exclusions**

Other diagnoses that do not share a common biological mechanism are best evaluated separately and only when clearly relevant to the treatment under investigation.

1. Pulmonary embolism.
2. Pleural effusion.
3. Cardiogenic pulmonary oedema.
4. Pneumothorax.
5. Bronchospasm.

Alt-text: Box 2

Box 3 **Secondary and exploratory outcomes**

**Secondary outcomes**

1. Death up to hospital discharge (or postoperative day 7 if still in hospital).
2. Atelectasis up to hospital discharge (or postoperative day 7 if still in hospital).
3. Pneumonia up to hospital discharge (or postoperative day 7 if still in hospital).
4. Acute respiratory distress syndrome up to hospital discharge (or postoperative day 7 if still in hospital)
5. Pulmonary aspiration up to hospital discharge (or postoperative day 7 if still in hospital).
6. Postoperative nausea and vomiting up to postoperative day 1.
7. Unplanned ICU/high-dependency unit admission up to hospital discharge.
8. Days alive and at home at 30 days.
9. Change in health-related quality of life (as measured by the EQ-5D-5L) between baseline and 3 months.

**Exploratory outcomes**

1. Duration of PACU stay.
2. Airway instrumentation between leaving the operating room and the end of the operative day.
3. Change in quality of recovery between baseline and postoperative day 1.
4. Change in frailty between baseline and 3 months.
5. Severity of postoperative pulmonary complications.

Alt-text: Box 3

### Further research

At least five further analyses of study data are planned, including: (1) sugammadex, neostigmine, and postoperative pulmonary complications, evaluating incremental health care costs and patient outcomes; (2) sugammadex, neostigmine, and postoperative nausea and vomiting; (3) frailty and postoperative pulmonary complications; (4) sugammadex, neostigmine, and 1-yr mortality (using additional data from registries); and (5) individual patient data meta-analysis of large randomised trials of sugammadex, neostigmine, postoperative pulmonary complications, and other outcomes.

### Sample size calculation

We anticipate that the incidence of postoperative pulmonary complications in this cohort will be around 16%. This is based on our Elimination of Nitrous Oxide in the Gas Mixture for Anaesthesia trial,^43^ in which the incidence of pneumonia, atelectasis, or both was 19.6% in patients aged ≥45 yr with cardiovascular risk factors having major noncardiothoracic surgery (unpublished data). It is also consistent with studies of upper abdominal surgery (20%),^2^ lower abdominal surgery (16%),^9^ and major abdominal surgery (19%).^6^

Our primary outcome is a composite of new pulmonary complications or death (estimated at <2% in this cohort^43^). We have chosen a 25% reduction in the incidence of new pulmonary complications or death (i.e. 16–12%) as a clinically meaningful effect size for this study and a finding that would be necessary to change practice. A study based on this effect size was estimated to require 1580 patients per group to achieve 90% power and assuming a two-sided 5% level of significance. We planned to recruit 3500 to account for ∼9% loss to follow-up (observed 0% up to hospital discharge [or postoperative day 7 if still in hospital] and 3.3% up to 3 months, 95% CI 0.9–8.3% in our pilot study^35^).

### Data analysis

The analysis and reporting of our results will comply with the Consolidate Statement of Reporting Clinical Trials (CONSORT) guideline.^44^ A detailed statistical analysis plan will be written before unblinding of the study data. This plan will describe all analysis principles and methods in detail, including primary and secondary treatment effects of interest and alternative models in the case of non-convergence or violation of underlying model assumptions. The primary outcome will be analysed using log-binomial regression, with the neostigmine group as the reference, and including in the model site and treatment to obtain the causal effect of the intervention assignment. It is anticipated that not all participants will receive the randomised intervention as planned (e.g. either no intervention or crossover between interventions [treatment switching]). The primary hypothesis will be evaluated by obtaining the estimate of the RR and a two-sided 95% CI along with a *P*-value. The model provides valid inference in the presence of missing data if the missing data mechanism is missing completely at random. A sensitivity analysis will be conducted using multiple imputation to explore the impact of any deviations from missing completely at random on the results.

Binary outcomes will be analysed in a similar manner to the primary outcome, and continuous outcomes will use a quantile or linear regression model with the outcome appropriately transformed before fitting the model if needed (e.g. skewed). Heterogeneity of treatment effect will be explored for subgroups (e.g. laparoscopic *vs* open surgery) independent of the study findings. Safety outcomes and adverse events will be summarised and intervention effects will be explored between the actual intervention groups.

### Interim analysis

One interim analysis was planned at the availability of the primary outcome in 50% of the patients. The interim analysis plan allowed stopping for superiority early using a conservative Haybittle–Peto rule with early stopping to reject the null hypothesis of no treatment effect. Consistent with the Haybittle–Peto approach, this interim analysis will have a negligible effect on the final α level and therefore the full two-sided α level of 0.05 was used in the planning of the trial and will be used in the final analysis.

### Patient and public involvement

Community representatives reviewed the protocol for this research and provide ongoing oversight as members of the trial steering committee. Participants will be provided with a plain language statement of the trial results on request, after the main trial results have been published.

## Ethics and governance

The protocol was approved by the Royal Melbourne Hospital Research Ethics Committee in Australia on 27 March 2023 (HREC/93017/MH-2023). The protocol was then approved by ethics committees in Aotearoa New Zealand and Hong Kong and by the research governance offices at each participating site in all three regions. Investigators and trial coordinators obtain written informed patient consent before surgery. A unique study number is assigned to each patient. Records are secured in locked filing cabinets and on password-protected computers, which are only accessed by approved study staff. Results from the study will be published in medical journals and will be presented at scientific meetings. The protocol was registered on the Australian New Zealand Clinical Trials Registry on 18 April 2023 (ACTRN 12623000394640). The current version of the protocol is version 5.0 dated 18 November 2025. The protocol and statistical analysis plan will be made publicly available. The trial steering committee will oversee data sharing in line with the trial data sharing statement in the trial registration.

## Declaration of interests

The authors have no conflicts of interest to declare.

## Authors’ contributions

Study conception and design: all authors

Wrote the first draft of the protocol: KL and SB

Critical revision of the draft for important intellectual content and content relevant to participants and the community: all authors

Guarantor: KL

Read and approved of the final version of the manuscript to be published: all authors

## Funding

Australian Medical Research Future Fund (APP2022850); Hong Kong Health and Medical Research Fund (11220226); 10.13039/501100001782University of Melbourne.
